# Loss of Function SETD2 Mutations in Poorly Differentiated Metastases from Two Hürthle Cell Carcinomas of the Thyroid

**DOI:** 10.3390/cancers12071892

**Published:** 2020-07-14

**Authors:** Valeria Pecce, Antonella Verrienti, Luana Abballe, Raffaella Carletti, Giorgio Grani, Rosa Falcone, Valeria Ramundo, Cosimo Durante, Cira Di Gioia, Diego Russo, Sebastiano Filetti, Marialuisa Sponziello

**Affiliations:** 1 Department of Translational and Precision Medicine, “Sapienza” University of Rome, 00161 Rome, Italy; valeria.pecce@uniroma1.it (V.P.); antonella.verrienti@uniroma1.it (A.V.); luana.abballe@uniroma1.it (L.A.); giorgio.grani@uniroma1.it (G.G.); rosa.falcone@uniroma1.it (R.F.); valeria.ramundo@uniroma1.it (V.R.); cosimo.durante@uniroma1.it (C.D.); sebastiano.filetti@uniroma1.it (S.F.); 2 Department of Radiological, Oncological and Pathological Sciences, “Sapienza” University of Rome, 00161 Rome, Italy; raffaella.carletti@uniroma1.it (R.C.); cira.digioia@uniroma1.it (C.D.G.); 3 Department of Health Sciences, “Magna Graecia” University of Catanzaro, 88100 Catanzaro, Italy; d.russo@unicz.it

**Keywords:** SETD2 loss-of-function mutations, Hürthle cell carcinoma, poorly differentiated thyroid cancer

## Abstract

Hürthle cell carcinomas (HCC) are rare differentiated thyroid cancers that display low avidity for radioactive iodine and respond poorly to kinase inhibitors. Here, using next-generation sequencing, we analyzed the mutational status of primary tissue and poorly differentiated metastatic tissue from two HCC patients. In both cases, metastatic tissues harbored a mutation of SETD2, each resulting in loss of the SRI and WW domains of SETD2, a methyltransferase that trimethylates H3K36 (H3K36me3) and also interacts with p53 to promote its stability. Functional studies of the novel p.D1890fs6* mutation (case 1) revealed significantly reduced H3K36me3 levels in SETD2-mutated tissue and primary cell cultures and decreased levels of the active form of p53. Restoration of SETD2-wildtype expression in the SETD2-mutant cells significantly reduced the expression of four well-known stemness markers (OCT-4, SOX2, IPF1, Goosecoid). These findings suggest potential roles for SETD2 loss-of-function mutations in HCC progression, possibly involving p53 destabilization and promotion of stemness. Their prevalence and potential treatment implications in thyroid cancer, especially HCC, require further study.

## 1. Introduction

Hürthle cell carcinoma (HCC) is a rare type of well-differentiated thyroid cancer derived from the follicular cells of the gland. It accounts for approximately 2% of thyroid cancer diagnoses [1]. Hürthle cells are present in thyroid adenomas and thyroid carcinomas [2]. The latter diagnosis is made when capsular and/or vascular invasion are present [3]. The distinction between Hürthle cell adenoma, minimally invasive HCC (HMIN), and widely invasive HCC (HWIDE) is crucial in terms of clinical outcomes. Hürthle cell adenomas (HCAs), which are benign, are readily cured by thyroid lobectomy, and HMINs exhibit an indolent course and very rarely produce lymph-node or distant metastases. In contrast, HWIDEs are more locally invasive and frequently metastasize to regional lymph nodes and distant metastatic sites, including the lung and bone [4].

In general, HCCs typically display low avidity for radioactive iodine (RAI) and poor responses to chemotherapy and radiation. This is particularly true of metastatic HWIDEs, which are almost always RAI-refractory and completely unresponsive to chemotherapeutic agents [5]. Standard therapy for RAI-refractory HCCs currently involves systemic treatment with multi-kinase inhibitors. The two Food and Drug Administration-approved agents currently used for managing metastatic progressive HCCs, lenvatinib, and sorafenib, display limited efficacy [6]. As a result, these tumors are associated with poorer outcomes than are HMINs or other differentiated thyroid cancers [5,7].

A recent study of 56 HCCs revealed mutational burdens that are higher than those of other differentiated thyroid cancers [8]. The somatic mutations identified in these HCCs affected the canonical signal transduction and tumor-suppressor pathways involved in thyroid cancer, including the RAS/RAF/MAPK and PI3K/AKT/mTOR cascades (55% of the tumors) and those involved in DNA damage and repair (38%). Non-silent mitochondrial DNA mutations were found in 35 (71%) of the 49 HCCs analyzed for such alterations [8]. Interestingly, 59% of the mutations found in HCCs were capable of altering the gene’s epigenetic profile, and the vast majority of these affected chromatin modification genes [8].

SETD2 is the sole methyltransferase involved in the trimethylation of lysine-36 of histone H3 (H3K36me3) in eukaryotic cells [9], an epigenetic mark associated with transcription activation and elongation [9,10]. The importance of H3K36me3 in the development of human cancer is well-documented. Relatively high frequencies of loss-of-function *SETD2* mutations have been reported in many tumor types, including acute lymphoblastic (10%) and myeloid leukemias (6%), adenocarcinoma of the lung (5%), and above all, clear cell renal cell carcinoma (15–20%), where SETD2 mutations have also been linked to advanced clinical stage at diagnosis and poor prognosis [11]. In addition, certain tumors harbor mutations involving the methyl acceptor site of H3K36 and adjacent amino-acid residues in histone H3.3, which also diminish H3K36me3 [12,13]. In thyroid cancers, however, loss-of-function *SETD2* mutations appear to be fairly rare; they were found in only 0.9% of the aggressive forms of thyroid cancer analyzed by Landa et al. [14].

The present study began with the observation of a case of HCC associated with poorly differentiated distant metastases that were RAI-refractory and displayed primary resistance to multi-kinases inhibitors (KIs). In an attempt to identify druggable targets in these lesions, we undertook an in-depth analysis of the mutational profiles of the primary and metastatic tumor tissues, which revealed a novel *SETD2* mutation whose molecular effects were documented by in vitro studies. Several years later, we encountered a second case of HCC that was strikingly similar to the first in terms of its clinical behavior. To our surprise, analysis of the second patient’s poorly differentiated metastatic tumors revealed that the mutational profiles of the two tumors were also quite similar.

## 2. Results

### 2.1. Next-Generation Sequencing (NGS) and Oncoscan Analysis of Patient 1′s Primary HCC and Poorly Differentiated Metastases

In brief, Patient 1 was 50-year-old women who underwent total thyroidectomy for HCC and subsequently developed locoregional and liver metastases that were both RAI- and KI-resistant. Sanger sequencing of primary and metastatic tissues ruled out the presence of any mutations in known thyroid-cancer–related genes (*H-, K-, N-RAS, BRAF, PTEN, PIK3CA*), and PAX8-PPARG fusions were excluded by RT-PCR. (See Methods for further details.)

**Figure 1:**
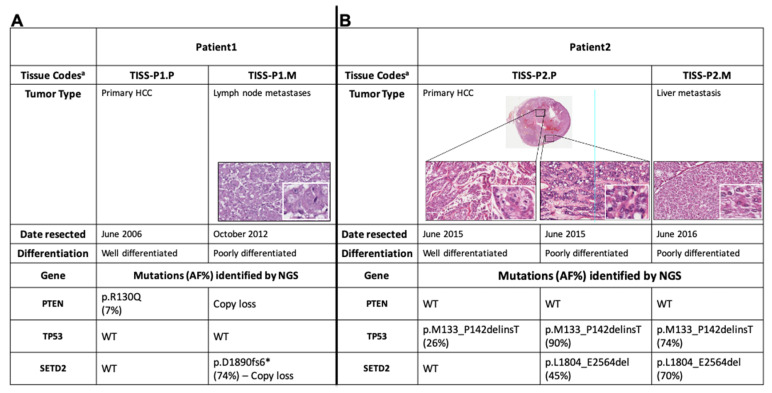
Histological and molecular features of the tumor tissues analyzed from (**A**) case 1 and (**B**) case 2. ^a^ All four tissues were Sanger sequencing-negative for mutations in *H-, K-, and NRAS; BRAF, PTEN, PI3KCA* and RT-PCR-negative for PAX8-PPARG. AF, allele frequency; NGS, next-generation sequencing; RT-PCR, reverse-transcription polymerase chain reaction.

NGS of the coding regions of 409 tumor-suppressor genes and oncogenes revealed 12 somatic variants in Patient 1′s primary tumor tissue (TISS-P1.P in Figure 1) and 5 in her lymph-node metastasis (TISS-P1.M) (Table S1). As shown in Figure 1A, TISS-P1.P harbored a well-known *PTEN* mutation (p.R130Q, COSM5033), which had escaped detection by Sanger sequencing because of its low allele frequency (AF, 7%). The presence of this PTEN mutation was confirmed by NGS of the same tissue with a smaller custom panel at higher sequencing coverage (average base coverage depth, 1122x; AF, 6%). The same tissue also displayed multiple macro-deletions involving chr2q37.3 (specifically, the PDCD1 locus, which encodes a negative regulator of antitumor immunity), chr22.p11, and chr8q11 (homozygous in the last chromosome). Duplications were also found involving chromosomes 7, 12, 16, and 18 (for details, see Table S2).

TISS-P1.M harbored the same chr2 and chr8 copy losses found in TISS-P1.P, as well as a novel heterozygous copy gain in chr7 that encompassed the *BRAF* locus (Table S2). The absence in TISS-P1.M of the PTEN mutation found in the primary tumor can be attributed to a homozygous copy loss identified in chromosome 10, which affected the region containing *PTEN* (and the *KLLN* locus, which encode a p53-regulated DNA replication inhibitor).

TISS-P1.M also displayed a novel loss-of-function mutation in the *SETD2* gene (p.D1890fs6*, AF: 46%) and a heterozygous copy loss of the chromosomal region containing *SETD2*. The fact that two *SETD2* alleles were found in TISS-P1.M was thought to reflect the high prevalence of stromal cells in the specimen (approximately 40% of all cells, as documented by histological review). To minimize the stromal contamination, we isolated a new DNA sample from a macrodissected fragment of TISS-P1.M and subjected it to NGS analysis at higher coverage (1433x) using a smaller custom panel that included *SETD2* as well as all the well-known thyroid-cancer-related genes. The results confirmed the existence of the *SETD2* mutation (p.D1890fs6*) at an AF of 74%, substantially higher than that in the undissected tissue, suggesting that its presence was limited exclusively to the tumor tissue. Re-analysis of the undissected tissue with the new NGS panel at higher coverage (1232x) confirmed the presence of *SETD2* mutation at a lower AF (47%). *SETD2* p.D1890fs6* affects exon 12 of the gene and generates a premature stop codon that results in the synthesis of a truncated SETD2 protein lacking two key C-terminal domains: the WW domain, which is involved in SETD2′s interaction with TP53, and the SRI domain, which interacts with the phosphorylated C-terminal domain of RNA polymerase II.

These findings prompted us to take a closer look at the possible roles played by SETD2 in the aggressiveness and progression of poorly differentiated thyroid cancers.

### 2.2. Effects of the SETD2 p.D1890fs6*Mutation on H3K36me3 Levels

*SETD2* encodes the histone methyltransferase responsible for H3K36me3. To explore the effects on this process of the *SETD2* p.D1890fs6* mutation, we first used Western blot analysis to assess SETD2 and H3K36me3 levels in TISS-P1.M and the primary cell line derived from this tissue.

**Figure 2:**
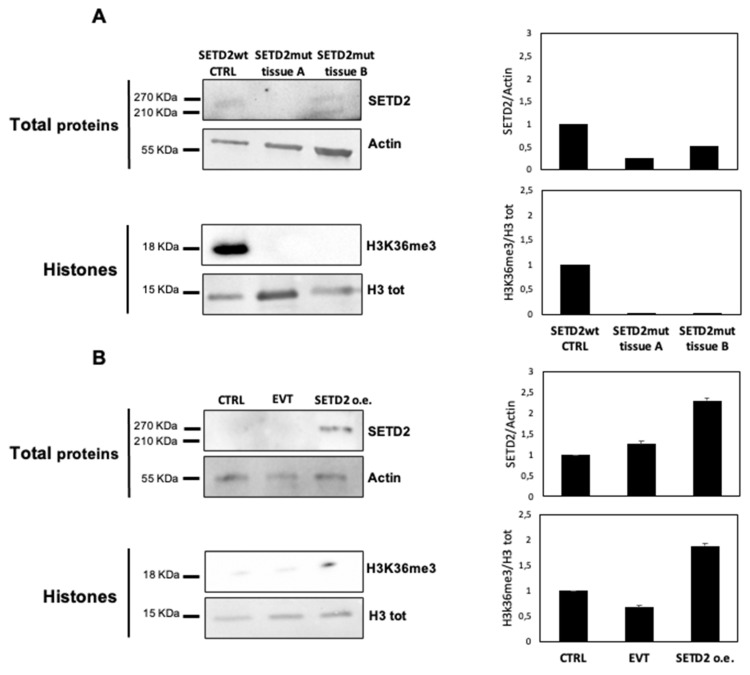
SETD2 protein and H3K36me3 levels are reduced in SETD2mut tissue and cells. Western blots (left-hand panels) with corresponding densitometric analyses (right-hand panels) are shown for SETD2, H3, and H3K36me3 levels in each of the following: (**A**) TISS-P1.M portions A and B and the SETD2-wildtype PDTC used as a control (SETD2-wt CTRL); (**B**) TISS-P1.M-derived primary cell cultures before (CTRL) and after transfection with empty vector (EVT) or vector containing SETD2-wildtype transcript (SETD2 o.e.). Actin and H3 were used, as indicated, for loading controls. Error bars in the densitometry graphs shown in panel B represent the standard deviation of the biological replicates (*n* = 3) included for this experiment.

Given the intratumoral heterogeneity of the primary lesion, we analyzed two different portions of fresh-frozen TISS-P1.M (SETD2mut tissues A and B) and compared the results to those obtained in poorly differentiated thyroid cancer (PDTC) harboring wild-type *SETD2*. As shown in Figure 2A, the SETD2-wt control tissue displayed a 270-KDa band corresponding to the full-length SETD2 protein and detectable levels of H3K36me3. In SETD2mut tissues A and B, H3K36me3 levels were undetectable, but SETD2 protein expression levels differed in the two tissue portions. In SETD2mut tissue A, SETD2 protein was absent, whereas in portion B, two bands were observed: a higher-molecular-weight band corresponding to the wild-type SETD2 protein and a lower-weight band representing the truncated protein encoded by *SETD2* p.D1890fs6*.

Next, we analyzed H3K36me3 levels in the cell lines derived from TISS-P1.M (SETD2mut cells), which were homozygous for the *SETD2* p.D1890fs6* mutation, as confirmed by Sanger sequencing. As shown in Figure 2B, these cells displayed no evidence of SETD2 protein expression or H3K36me3. To assess the effects of restored SETD2 expression, we transfected the SETD2mut cells with a vector containing wild-type SETD2cDNA under the control of a constitutive promoter (SETD2 o.e.) or with an empty vector (entire membranes are shown in Figure S1). As shown in Figure 2B, 48 h after transfection, SETD2 protein expression, and H3K36me3 levels were detectable only in the SETD2 o.e. cells. These cells also displayed a slight but statistically nonsignificant decrease in growth, as compared with empty vector-transfected controls (Figure S2).

**Figure 3:**
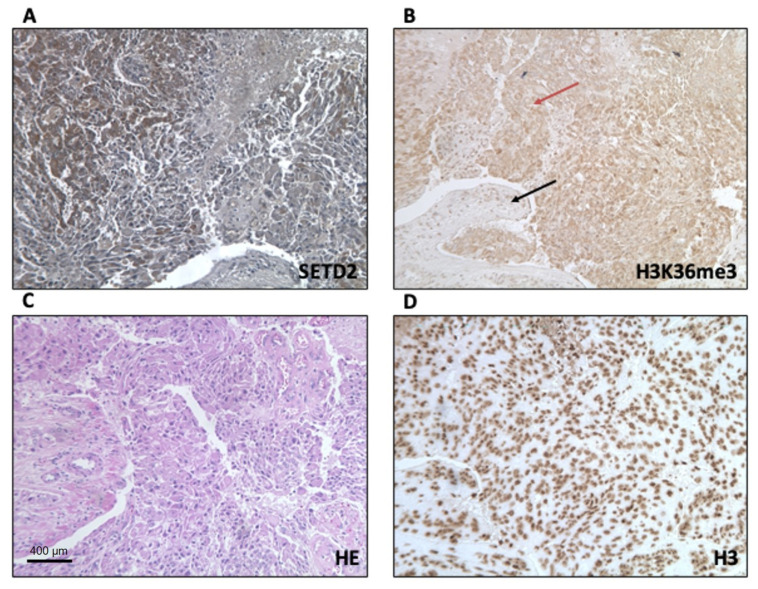
IHC analysis of the subcellular localization of SETD2 and H3K36me3 in SETD2mut TISS-p1.M tissue. (**A**) SETD2 protein expression; (**B**) trimethylation of lysine 36 of histone (black and red arrows indicate stromal and tumor cells, respectively); (**C**) hematoxylin-eosin (HE) staining of a representative section of TISS-P1.M; (**D**) total histone H3 protein expression, panel D (20×). Scale bar: 400 µm.

Next, we used immunohistochemistry (IHC) to analyze the expression levels and subcellular localization of SETD2 and H3K36me3 proteins in TISS-P1.M sections. SETD2 expression in the neoplastic cells was moderately intense and confined largely to the cytoplasm, with only a few positive nuclei. In the stromal cells, immunostaining was weak and localized to the nuclei (Figure 3A). Immunostaining for H3K36me3 in the tumor cells was also moderately intense and cytoplasmic, whereas stromal cells displayed strong, nuclear expression of H3K36me3 (Figure 3B). Histone H3 protein immunostaining was strong, localized in the nucleus, and homogeneously distributed in the tumor and stroma cells (Figure 3D). Results for non-neoplastic control tissues (positive controls suggested by the antibodies manufacturer) are shown in Figure S3.

**Figure 4:**
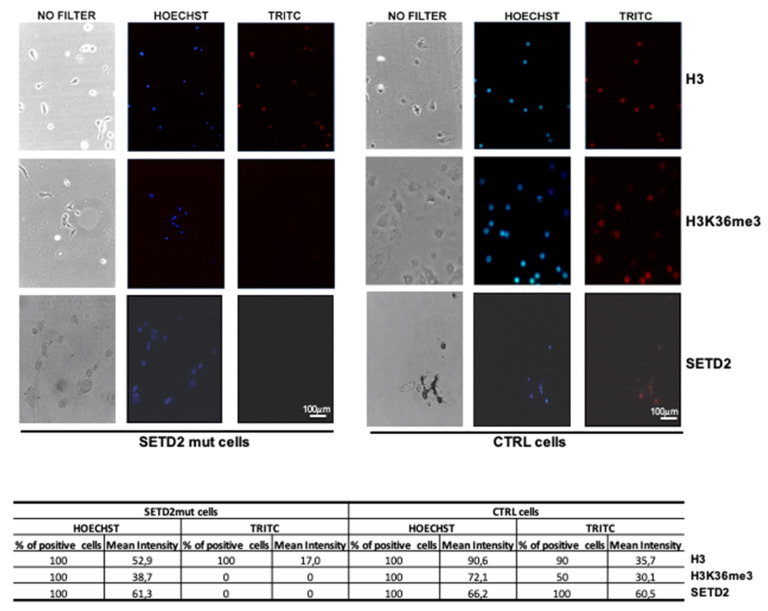
Immunofluorescence analysis of SETD2, H3, and H3K36me3 expression in TISS-P1.M-derived primary cell cultures (SETD2mut cells). Antibodies used to detect H3, H3K36me3, and SETD2 protein were tetramethylrhodamine (TRITC) dye-conjugated; nuclei were Hoechst-stained. SETD2mut cells were compared with a cell line established from an archived follicular proliferation used as SETD2-wt controls (CTRL). Fluorescent spots were quantified with Image J software (https://imagej.nih.gov/ij/), as reported in the tables below the images. Scale bar: 100 µm.

Immunofluorescence was then used to analyze the primary SETD2mut cell cultures. As shown in Figure 4, SETD2 and H3K36me3 expression levels were markedly lower than those observed in SETD2-wt controls. The absence of SETD2 activity, as reflected by H3K36me3 levels, in the nuclei of the mutant cells was confirmed by Western blot analysis of nuclear and cytoplasmic fractions (Figure S4).

Taken together, the results presented above indicate that the SETD2 p.D1890fs6* protein, which lacks the SRI and WW domains, is associated with considerably reduced H3K36me3 activity.

### 2.3. Reduced Stability of Active Forms Of P53 in SETD2mut Samples

**Figure 5:**
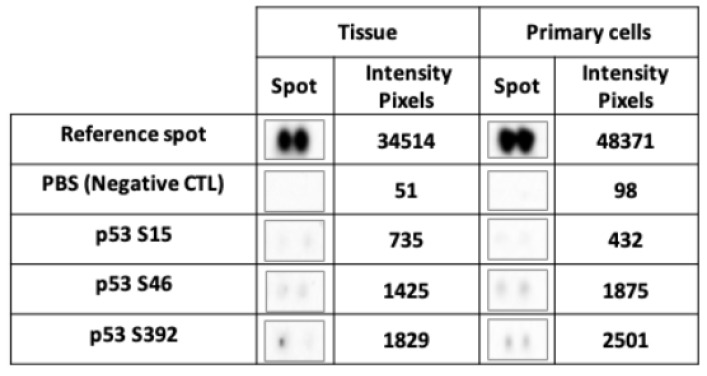
Instability of phosphorylated TP53 protein in SETD2mut tissue and cells. Levels of phosphorylated serines 15, 46, and 392 of TP53 protein detected with a commercial human phosphoprotein array. The reference spot was the positive control; PBS was the negative control. Phosphorylation levels were quantified using ImageLab software (BioRad). The reported pixel intensity is the mean obtained for the two spots. Positivity was defined as an intensity of >3000 pixels, as recommended by the software manufacturer.

One of the functions of SETD2 is to stabilize the active form of p53. SETD2 has been shown to interact with p53′s transactivation domain (TAD) [15], which binds HDM2, an ubiquitin-protein ligase that inhibits the tumor suppressor’s activity and promotes its degradation. The stability of p53 can be augmented in two ways: via p53 phosphorylation at serine 15, which causes its dissociation from HDM2, and/or by SETD2-mediated inhibition of HDM2 expression. To investigate the possible impact of the *SETD2* p.D1890fs6* mutation on p53 stability, we analyzed phosphorylation levels at p53 serines 15, 46, and 392 in TISS-P1.M and the cell cultures derived from this tissue. Despite high total p53 protein expression levels (Figure S5), the SETD2mut tissues and cells both displayed very low p53 phosphorylation levels (Figure 5), particularly at serine 15. These findings suggest that the p.D1890fs6* variant does indeed compromise SETD2′s ability to stabilize p53.

### 2.4. Restoration of SETD2 Expression Reduces the Stemness of SETD2mut Cells

**Figure 6:**
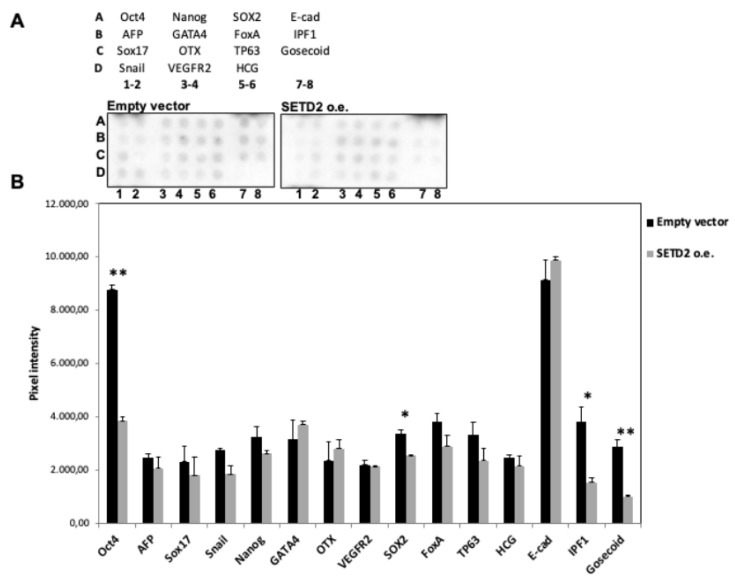
Impact of restored SETD2 expression on stemness markers in SETD2mut cells. Cells were tested 48 h after transfection with empty vector (controls) or the wild-type SETD2 construct (SETD2 o.e.). Cell lysates were incubated overnight on the array. (**A**) Diagram showing the positions in the Human Pluripotent Stem Cell Array of the 15 stemness proteins tested. The lower panels show array images after chemiluminescence acquisition (ImageLab, Biorad). In the array, each protein is represented in duplicate. (**B**) Pixel intensity (mean of the two spots) for each protein analyzed with the array. Significant differences by t-test: * *p* < 0.05, ** *p* < 0.005).

In several tumor types, de-differentiation is associated with a worse prognosis. Given the de-differentiation observed in TISS-P1.M, we decided to analyze the effect of restored wild-type SETD2 expression on the “stemness” of SETD2mut cells. Using a commercial protein array, we assessed the expression levels of 15 well-known stemness protein markers in SETD2 o.e. and EVT cells. As shown in Figure 6, the expression of four major stemness proteins (OCT-4, SOX2, IPF1, Goosecoid) was significantly reduced by the restoration of SETD2 wildtype expression, thereby suggesting that the *SETD2* p.D1890fs6* mutation plays a role in the de-differentiation process.

### 2.5. NGS Analysis of Primary Tumor and a Poorly Differentiated Metastasis from a Second HCC Patient

Several years after the death of Patient 1, we observed a second case of HCC with very similar clinical features (see Methods for details). To determine whether the clinical resemblance reflected similarities between the mutational profiles of the two patients’ HCCs, we analyzed primary and metastatic tumor tissues from the second patient (TISS-P2.P and P2.M) using the custom NGS panel that included *SETD2* and several well-known thyroid cancer-associated genes [16,17].

As shown in Figure 1B, the liver metastasis resected roughly one year after the initial thyroidectomy (TISS-P2.M) displayed a clear loss of differentiation. In contrast, the primary HCC displayed heterogeneity in terms of cell differentiation, with high-level differentiation in central sections and a clear loss of differentiation in sections taken near the border of the tumor. Both areas of TISS-P2.P and the poorly differentiated cells of TISS-P2.M harbored a non-frameshift mutation in *TP53* (p.M133_P142delinsT), with AFs in the poorly differentiated tissues that were markedly higher than that observed in the well-differentiated portion of the primary tumor. As for *SETD2*, it was found to be wild-type in the differentiated portions of the TISS-P2.P sample. However, a loss-of-function mutation (*SETD2* p.L1804_E2564del) was identified in the poorly differentiated cells at the primary tumor’s border and—with an even higher AF—in the poorly differentiated metastatic lesion removed from the liver (Figure 1B). The *SETD2* mutation identified in this case, like the p.D1890fs6* mutation identified in patient 1′s HCC, affected exon 12 of the gene, generated a premature stop codon, and resulted in the synthesis of a truncated SETD2 protein lacking the WW and SRI domains.

## 3. Discussion

H3K36 methylation plays roles in numerous nuclear pathways that are dysregulated in cancer (e.g., those involving transcriptional regulation, alternative splicing, DNA repair [18]), and the H3K36 methylation status of a gene is an important check to obtain the correct transcript. In human cells, H3K36me3 is carried out exclusively by SETD2, and it is required for preventing intragenic transcription initiation in 11% of human genes [19].

In several types of cancer (breast, renal, gastrointestinal stromal tumors, acute leukemia), underexpression and mutation of *SETD2* are associated with a poor prognosis [18]. In the cells of other types of tumors, loss-of-function *SETD2* mutations cause defects involving DNA repair, nucleosome dynamics, RNA processing, and DNA methylation [20]. We suspected that loss of SETD2 function might also be relevant in the de-differentiation of thyroid cancer. *SETD2* mutations appear to be rare in primary thyroid cancers: they were found in one (1.8%) of the 56 HCCs studied by Ganly et al. [8] and one (0.9%) of the 117 PDTC-ATCs investigated by Landa et al. [14].

However, no data are available on the frequency of *SETD2* mutations in thyroid cancer metastases, where the two *SETD2* loss-of-function mutations described in this report were found. And in both cases, the metastases were characterized by a clear loss of differentiation. In the second case, the mutation was also detected in a spatially restricted area of poorly differentiated tissue located in the periphery of the primary HCC. The remainder of the primary tumor was well-differentiated and *SETD2*-wt.

All of Patient 2′s tumor tissues also harbored a *TP53* gene mutation whose allelic frequency, like that of the *SETD2* mutation, increased as tumor cell differentiation diminished. Patient 1′s primary and metastatic tumors were both negative for *TP53* mutations, but one of the chromosomal copy losses found in TISS-P1.M involved a region that includes the *PTEN* gene. This copy loss explains the absence in the metastatic tissue of the *PTEN* mutation, the only known thyroid cancer-related driver mutation found in the patient’s primary HCC (at a very low frequency). The same copy loss included the *KLLN* gene, which encodes a DNA-binding protein involved in S phase checkpoint control-coupled apoptosis. *KLLN*, which mediates p53-induced apoptosis, is considered the link between p53 activation and the S phase checkpoint control designed to eliminate replicating precancerous cells. The absence of this protein leads to the accumulation of precancerous cells [21].

Our in vitro studies of SETD2mut tissues and cells from case 1 revealed aberrancies in both involving two of the more essential functions attributed to the SETD2 protein, namely, the trimethylation of lysine 36 of histone H3 and stabilization of the active form of p53. Neither of the *SETD2* mutations we observed functionally compromised the protein’s SET catalytic domain. Both mutations affected exon 12 of the gene, generated a premature stop codon, and resulted in the synthesis of a truncated SETD2 protein lacking the WW and SRI domains. Although in vitro functional studies of Patient 2′s mutation were not possible because fresh tissues were not available in this case, the fact that this mutation lacked the same domains as those lost in case 1 suggests that the two mutations might also have similar molecular effects.

In the first case, the loss of the WW and SRI domains caused a substantial reduction in the trimethylation signal in the SETD2mut tissue and its complete loss in the SETD2mut cells. Moreover, analysis of the mutant tissue revealed that SETD2 expression and trimethylation signals were both confined to the cytoplasm, possibly due to the loss of nuclear localization sequences in the SETD2-mutated protein. The fact that the *SETD2* mutation was homozygous in the primary SETD2-mut cell cultures probably reflects the absence in this cell population of contamination by other cell types present in the SETD2mut tissue.

Previous studies [15] have shown that the stability of the active form of p53 depends on binding between the WW domain of SETD2 and the TAD region of p53. This finding is fully consistent with our finding that active p53 is destabilized in the absence of a wild-type form of SETD2 in the thyroid cancer cells. Our data also suggest that SETD2 contributes to the de-differentiation of thyroid cancer cells, as reflected by the downregulated expression of four stemness proteins observed after wild-type SETD2 expression was restored in SETD2mut cells. A direct association has been previously found between the expression levels and the H3K36me3 status of the promoter regions of several pluripotency-associated genes, including SOX2 and OCT-4 [22], two of the four stemness proteins whose expression was dysregulated in SETD2mut cells. No data are available on the relationship between H3K36me3 and the expression of the other two stemness genes pinpointed by our experiments.

## 4. Materials and Methods

All subjects gave their informed consent for inclusion before they participated in the study. The study was conducted in accordance with the Declaration of Helsinki, and the protocol was approved by the Ethics Committee of Azienda Universitaria Policlinico Umberto I of Rome (Project identification code Prot. 1184/17).

### 4.1. Cases Analyzed

Patient 1 was a 50-year-old woman diagnosed in June 2006 with a widely invasive HCC that was treated with total thyroidectomy. The tumor (maximum diameter: 45 mm) was staged as pT3 Nx, according to the criteria of the AJCC TNM classification, 7th edition. After surgery, she also received radioactive iodine (RAI) therapy. Over the next three years, she developed cervical lymph-node metastases and liver lesions. The latter were treated in 2009 with a left liver lobectomy and the lesions histologically confirmed as HCC metastases. In 2010, a second cycle of RAI therapy was administered (cumulative activity 250 mCi) for disease progression. A few months later, PET/CT scan revealed ongoing progression of the liver disease, with increases in both the number and size of lesions. The patient was enrolled in a phase 3 clinical trial of sorafenib, but treatment was discontinued nine months later because the liver disease had progressed and new bone and lymph node lesions were documented. In November 2011, second-line therapy with sunitinib was started, but it was discontinued one year later because of a serious adverse event. Meanwhile, the progression of the neck disease had begun to cause tracheal compression associated with moderately severe dyspnea, which was treated palliatively with a lateral neck dissection in October 2012. Histological examination of the resected tissue confirmed the diagnosis of a poorly differentiated thyroid cancer, and the patient died a few months later.

Patient 2 was a 56-year-old woman referred to our hospital in 2018. In 2015, she had undergone total thyroidectomy for a Hürthle cell carcinoma (stage pT2, AJCC 7th edition). One year later (June 2016), a CT scan revealed a single liver lesion suspected to be metastatic. A wedge resection of the right liver lobe was performed, and the histological diagnosis was poorly differentiated Hürthle cell carcinoma. In October 2016, RAI was administered (150 mCi) because of progressing bone and liver disease, but two months later, the lesions were judged to be RAI-refractory and further progression was documented. Lenvatinib was started and continued for 15 months with disease stabilization as the best response. In March 2018, when first seen by our staff, a CT scan showed additional progression in the lung, liver, bone, spleen, and lymph nodes, and second-line treatment with sorafenib was started. The drug had no effect on the rapidly-spreading disease. The patient’s clinical condition deteriorated over the next few months, and she died in August 2018.

Unless otherwise stated, all commercial products were used in accordance with datasheets provided by the manufacturer.

### 4.2. Biospecimens Analyzed

A limited number of patient-derived specimens from the two cases were available for analysis. As summarized in Figure 1A,B, they included: preoperative peripheral venous blood samples from patients 1 and 2; four formalin-fixed paraffin-embedded (FFPE) tumor tissues (primary HCCs from patient 1: [TISS-P1.P] and patient 2 [TISS-P2.P]; metastatic lesions from patient 1 [TISS-P1.M] and patient 2 [TISS-P2.M]); and a fresh-frozen sample of TISS-P1.M. Two different pathologists confirmed the original diagnoses of all tissue specimens submitted for standard histopathological examination.

#### 4.2.1. Cell Cultures

Primary tumor-cell cultures were established, as previously described [23], from the fresh frozen specimens of TISS-P1.M and an archived follicular proliferation (used as a SETD2-wildtype (WT) control in immunofluorescence experiments). Cultures were subjected to partial trypsinizations to enrich their tumor-cell contents [24], and after the first passage, they were treated with magnetic anti-fibroblast beads (Miltenyi Biotec GmbH, Bergisch Gladbach, Germany) to further reduce stromal-cell contamination. HeLa CCL-2 cells (used as SETD2-WT controls in western blot experiments) were cultured in DMEM medium (Gibco-BRL Division, Thermo Fisher Scientific, Waltham, MA, USA) containing 10% FBS (Gibco-BRL Division, Thermo Fisher Scientific) and Antibiotic–Antimycotic solution (Gibco-BRL Division, Thermo Fisher Scientific) and incubated at 37 °C in an atmosphere of 5% CO_2_.

#### 4.2.2. Nucleic Acid Isolation

DNA was isolated from FFPE tumor tissues using the NucleoSpin Tissue Kit (Macherey-Nagel GmbH & Co. KG, Düren, Germany) and from peripheral blood with the QIAmp-Blood MIDI Kit (Qiagen, Hilden, Germany). Total RNA was isolated from fresh-frozen tumor tissues using Trizol reagent (Thermo Fisher Scientific). The RNeasy Mini Kit (Qiagen, Hilden, Germany) was used to isolate total RNA from cell cultures. DNA and RNA concentrations were measured with a NanoDrop Spectrophotometer and Qubit^®^ quantification assays (both from Thermo Fisher Scientific). First-strand complementary DNA (cDNA) was synthesized using the High Capacity cDNA Reverse Transcription kit (Thermo Fisher Scientific).

### 4.3. Next-Generation Sequencing (NGS)

Forty nanograms of DNA from patient 1′s blood, primary and metastatic tumor tissues (Figure 1A) were analyzed on an Ion Torrent PGM instrument with the Ion AmpliSeq™ Comprehensive Cancer Panel (CCP) (Thermo Fisher Scientific), which simultaneously interrogates the coding sequences of over 400 oncogenes and tumor suppressor genes. Following targeted amplification, amplicons were partially digested, phosphorylated, and ligated to ion adapters. Each library was enriched by clonal amplification on an Ion One Touch2 System. Finally, sequencing was performed using Ion PGM Sequencing 200 kit v.2 on two Ion 318 chips, as previously described [25]. Data were analyzed as previously described [25]. Briefly, we used Torrent Suite software v.4 with the Coverage and the Variant Caller plugins and somatic-low stringency parameters optimized for low-frequency variant detection (ThermoFisher Scientific). Variant caller format (VCF) files were then annotated with Ion Reporter 4.0 (Thermo Fisher Scientific) and wANNOVAR software. Variants were called when a position was covered at least 100 times. The clinical sensitivity of point mutations and indels was set at 5% for known thyroid-cancer-related mutations (hotspots) and at 10% for any other variant. Variants were filtered based on their frequency among the European-descendent population, as reflected by a minor allele frequency of <0.005. Variants classified by ClinVar as “not-pathogenic”, “probable-not-pathogenic”, “drug response”, or “other” were excluded, as were intronic and synonymous variants. Final high-quality variants were those with a depth of coverage (DP) of 100, genotype quality (GQ) scores of 30, a minimum alternate allele frequency of 10% (AF = 10%), strand bias in variant relative to reference between 0.3 and 0.7, and absence of homopolymer regions (HRUN < 6).

The primary and metastatic tumor tissues from patient 2 (Figure 1B) were analyzed on the Ion GeneStudio S5 system with a custom multi-gene NGS panel, as previously described [16,17,26], starting from 15 ng of DNA and 10 ng of RNA. The panel tested for single-nucleotide variants/small indels (DNA panel) and gene fusions (RNA panel) involving SETD2 as well as 22 genes with documented relations to thyroid cancer (including all known thyroid cancer-related gene fusions) [16,17]. The assay uses an amplification-based approach for targeted enrichment of genomic regions. It was developed to generate short amplicons with a maximum length of 175 bp, which enables the analysis of degraded sample sources such as FFPE (AmpliSeq, ThermoFisher Scientific). Primary and metastatic tissues (macro-dissected and undissected portions of the metastases) from patient 1 were re-analyzed with the same custom panel in order to confirm *PTEN* and *SETD2* mutations previously detected. Data were analyzed using Torrent Suite v.10 software with the Coverage and Variant Caller plugins and somatic low stringency parameters (ThermoFisher Scientific). Ion Reporter v10 and wANNOVAR software were used for variant annotation. Variants were called when the position was covered at least 500 times. The clinical sensitivity of mutations was set at 5%. Variants were prioritized according to population frequency, functional consequences, quality values, as described above. RNA data were analyzed with Torrent Suite v.10 (ThermoFisher Scientific) and Ion Reporter 5.12 software (Thermo Fisher Scientific) using the workflow for gene-fusion detection.

#### 4.3.1. Sanger Sequencing

A standard Sanger sequencing protocol was used in the initial analysis of blood and tissue samples from patients 1 and 2 to detect variants in the H-, K-, N-RAS, BRAF, PTEN, PIK3CA genes. The standard Sanger sequencing protocol used to validate high-priority variants found during NGS analysis has been described in detail elsewhere [27]. PCR conditions and sequencing primers are available upon request.

#### 4.3.2. RT-PCR Analysis

Fifty nanograms of RNA from patients’ tissue samples were used to analyze PAX8-PPARgamma fusions, as previously described [28].

#### 4.3.3. Analysis of Single-Nucleotide Polymorphisms (SNPs)

TISS-P1.P and TISS-P1.M were evaluated for copy number variations (CNVs) using OncoScan^®^ arrays (Affymetrix, Santa Clara, CA, USA), which analyze 220,000 SNPs at carefully selected genomic locations. CNVs were computationally detected by comparison with a reference normal signal data set based on the standard protocol and analysis pipeline for Oncoarray [29].

### 4.4. Protein Extractions

Total proteins were extracted from fresh-frozen tissues (TISS-P1.P and an archived sample of SETD2-wild-type poorly differentiated thyroid cancer (PDTC) used as a control) and the pellets of cell-cultures (derived from TISS-P1.P and HeLa cells used as SETD2-wt controls) with a lysis buffer containing TrisHCl (pH 7.4, 50 mM), NaCl (150 mM), Triton (1% *v*/*v*), ethylenediaminetetraacetic acid (EDTA, 20 mM), phenylmethylsulfonyl fluoride (PMSF, 2 mM), protease and phosphatase inhibitors (Pierce, Rockford, IL, USA), leupeptin (2 ug/mL), glycerol (10% *v*/*v*). Histone samples were obtained by acidic extraction. Minced tissues and cell-culture pellets were suspended in HCl 0.5 M containing PMSF and protease and phosphatase inhibitors (Pierce, Rockford, IL, USA). The lysate was centrifuged at 2500× *g* for 10 min at 4 °C. The supernatant was collected and processed with the lysis buffer described above. HeLa cell and TISS-P1.P-derived cell culture pellets were resuspended in specific buffers to obtain the cytoplasmic and nuclear fractions of each. Two buffers were used to obtain the cytoplasmic fractions: lysis buffer A, which was composed of hydroxyethyl-1-piperazineethanesulfonic acid, pH 7.4 (HEPES, 20 mM), glycerol (20% *v*/*v*), KCl (50 mM), and EDTA (1 mM), and lysis buffer B, which consisted of 4-nonylphenyl-polyethylene glycol (NP-40), 10% *v*/*v*. Nuclear fractions were obtained with a single buffer (Lysis buffer C), which consisted of HEPES (20 mM), glycerol (25% *v*/*v*), NaCl (400 mM), and EDTA (2 mM). All extractions were done on ice and the products quantified using the Bradford method [30].

#### 4.4.1. Protein Expression Analyses

For western blot analysis, 30 μg of total proteins and 15 μg of histones were separated using SDS-PAGE and transferred onto polyvinyldene difluoride (PVDF) membranes. 50 μg of total proteins were then assayed with the Human Phospho-Kinase Antibody Array and Human Pluripotent Stem Cell Array (R&D Systems, Minneapolis, MN, USA).

For immunofluorescence (IF) analyses, cells were fixed in paraformaldehyde (PFA, 4%), permeabilized with phosphate-buffered saline (PBS, 1×) containing Triton X-100 (0.1%), and blocked with bovine serum albumin (BSA, 3%) in PBS. Primary and secondary antibodies were applied at 1:100 and 1:500 dilutions, respectively. Nuclei were stained with Hoechst 1:5000 in PBS 1×.

Immunohistochemical (IHC) studies were performed on consecutive 3 μm-thick sections of FFPE tumor tissue. The sections were deparaffinized in xylene, rehydrated through a graded alcohol series, and treated with 3% hydrogen peroxide to block endogenous peroxidase activity. Following an antigen retrieval step, which involved boiling in citrate buffer (0.01 mol/L, pH 6) in a microwave (750 W), the sections were incubated with the primary antibodies (anti-H3 and anti-H3K36me3, 1:300, for 1 h at room temperature or anti-SETD2, 1:50, overnight at 4 °C). The reaction was amplified with CRF Anti-Polyvalent HRP Polymer (Scyteck Laboratories, Logan, UT, USA) and the amplicon visualized with 3,3-diaminobenzidine (DAB) (Dako, Glostrup, Denmark). The sections were counterstained with Mayer’s hematoxylin. All analyses included negative controls prepared as described above but without the primary antibody. The IHC evaluation for each antibody was performed by 2 experienced investigators who analyzed the immunostaining in the entire tumor area present in the slide (at magnification 20×). Results were expressed with a semiquantitative double score based on staining localization (nuclear or cytoplasmic) and intensity (negative, weak, moderate, strong) [31].

Proteins were detected with rabbit polyclonal anti-H3 antibody (ab12149, Abcam, Cambridge, UK), rabbit polyclonal anti-H3 tri methyl K36 antibody (ab1785, Abcam), rabbit polyclonal anti-SETD2 antibody (LS-C8109, LSBio, Seattle, WA, USA), and mouse monoclonal anti-β-actin antibody (A5316, Sigma Aldrich, St. Louis, MO, USA). Histones, SETD2, β-actin, and phosphorylated p53 were detected using chemiluminescent western blotting with Clarity Western ECL substrate (BIO-RAD, Hercules, CA, USA) and a charge-coupled-device camera (Chemidoc, BIO-RAD).

#### 4.4.2. Transfection and Growth Analysis

Lipofectamine 3000 (Thermo Fisher Scientific) was used to transiently transfect SETD2mut cells with an empty pFN21A Halo Tag CMV vector or the same vector containing SETD2 transcript sequence (Promega). The day before the transfection, cells were transferred to 6 well-plates containing the culture medium described above (in Cell Cultures). Two hours before transfection, the cells were starved in DMEM/F12 medium without FBS, glutamine, or antibiotics. Cells were transfected and counted 24 h, 48 h, and 72 h after the transfection. The transfection was performed using 2 μg of plasmid for each well added to 5 μL of P3000 Reagent and 5 μL of Lipofectamine 3000 Reagent.

### 4.5. Statistical Analysis

Data are reported as means ± standard deviation. Differences observed under different conditions or in different samples were assessed with an unpaired *t*-test, and *p*-values lower than 0.05 were considered statistically significant. All statistical analyses were performed using GraphPad Prism version 5.0 statistical software (GraphPad Software Inc., San Diego, CA, USA).

## 5. Conclusions

In conclusion, we documented the presence of two loss-of-function mutations of the histone methyltransferase SETD2 in the poorly differentiated metastatic tissues from two widely invasive HCC. Further investigation is needed to improve our understanding of the mechanisms underlying the effects of H3K36me3 signal in cancer cells. However, a WEE1-kinase inhibitor, AZD1775, has recently displayed efficacy in the treatment of H3K36me3-deficient tumors [32] and is currently undergoing phase I testing in patients with advanced TKI-refractory solid tumors (https://clinicaltrials.gov/ct2/show/record/NCT01748825). In light of the results that emerged from our study, SETD2 appears to be a novel and potentially druggable target for the management of thyroid cancers, in particular those with poor prognoses.

## Supplementary Materials

The following are available online at https://www.mdpi.com/2072-6694/12/7/1892/s1, Figure S1: Entire membranes shown in Figure 2, Figure S2: Growth of SETD2mut cells before and after transfection, Figure S3: Control tissues used in IHC studies of SETD2mut TISS-P1.M, Figure S4: Western blot and densitometric analysis of H3K36me3 in cytoplasmic (C) and nuclear (N) fractions of SETD2mut cells, Figure S5: Total TP53 expression in SETD2mut tissue and cells, Table S1: Variants found in TISS-P1.P and TISS-P1.M, Table S2: Copy number variation results of TISS-P1.P and TISS-P1.M.

## References

[1] Fagin J.A., Wells S.A. (2016). Biologic and Clinical Perspectives on Thyroid Cancer. N. Engl. J. Med..

[2] Máximo V., Lima J., Prazeres H., Soares P., Sobrinho-Simões M. (2012). The biology and the genetics of Hürthle cell tumors of the thyroid. Endocr. Relat. Cancer.

[3] Ghossein R.A., Hiltzik D.H., Carlson D.L., Patel S., Shaha A., Shah J.P., Turtle R.M., Singh B. (2006). Prognostic factors of recurrence in encapsulated Hurthle cell carcinoma of the thyroid gland: A clinicopathologic study of 50 cases. Cancer.

[4] Ganly I., McFadden D.G. (2019). Short Review: Genomic Alterations in Hürthle Cell Carcinoma. Thyroid.

[5] Besic N., Vidergar-Kralj B., Frkovic-Grazio S., Movrin-Stanovnik T., Auersperg M. (2003). The Role of Radioactive Iodine in the Treatment of Hürthle Cell Carcinoma of the Thyroid. Thyroid.

[6] Grani G., Lamartina L., Durante C., Filetti S., Cooper D.S. (2018). Follicular thyroid cancer and Hürthle cell carcinoma: Challenges in diagnosis, treatment, and clinical management. Lancet Diabetes Endocrinol..

[7] Kushchayeva Y., Duh Q.Y., Kebebew E., Clark O.H. (2004). Prognostic indications for Hürthle cell cancer. World J. Surg..

[8] Ganly I., Makarov V., Deraje S., Dong Y.Y., Reznik E., Seshan V., Nanjangud G., Eng S., Bose P., Kuo F. (2018). Integrated Genomic Analysis of Hürthle Cell Cancer Reveals Oncogenic Drivers, Recurrent Mitochondrial Mutations, and Unique Chromosomal Landscapes. Cancer Cell.

[9] Edmunds J.W., Mahadevan L.C., Clayton A.L. (2008). Dynamic histone H3 methylation during gene induction: HYPB/Setd2 mediates all H3K36 trimethylation. EMBO J..

[10] Nam S.J., Lee C., Park J.H., Moon K.C. (2015). Decreased PBRM1 expression predicts unfavorable prognosis in patients with clear cell renal cell carcinoma. Urol. Oncol. Semin. Orig. Investig..

[11] Hakimi A.A., Ostrovnaya I., Reva B., Schultz N., Chen Y.B., Gonen M., Liu H., Takeda S., Voss M.H., Tickoo S.K. (2013). Adverse outcomes in clear cell renal cell carcinoma with mutations of 3p21 epigenetic regulators BAP1 and SETD2: A report by MSKCC and the KIRC TCGA research network. Clin. Cancer Res..

[12] Patnaik M.M., Abdel-wahab O. (2018). SETD2—Linking stem cell survival and transformation. Cell Res..

[13] Fahey C.C., Davis I.J. (2017). SETting the stage for cancer development: SETD2 and theconsequences of lost methylation. Cold Spring Harb. Perspect. Med..

[14] Landa I., Ibrahimpasic T., Boucai L., Sinha R., Knauf J.A., Shah R.H., Dogan S., Ricarte-Filho J.C., Krishnamoorthy G.P., Xu B. (2016). Genomic and transcriptomic hallmarks of poorly differentiated and anaplastic thyroid cancers. J. Clin. Investig..

[15] Xie P., Tian C., An L., Nie J., Lu K., Xing G., Zhang L., He F. (2008). Histone methyltransferase protein SETD2 interacts with p53 and selectively regulates its downstream genes. Cell Signal..

[16] Sponziello M., Brunelli C., Verrienti A., Grani G., Pecce V., Abballe L., Ramundo V., Damante G., Russo D., Lombardi C.P. (2020). Performance of a dual-component molecular assay in cytologically indeterminate thyroid nodules. Endocrine.

[17] Verrienti A., Pecce V., Abballe L., Ramundo V., Falcone R., Inanloo Nigi Jak F., Brunelli C., Fadda G., Bosco D., Ascoli V. (2020). Analytical validation of a novel targeted next-generation sequencing assay for mutation detection in thyroid nodule aspirates and tissue. Endocrine.

[18] Rogawski D.S., Grembecka J., Cierpicki T. (2016). H3K36 methyltransferases as cancer drug targets: Rationale and perspectives for inhibitor development. Future Med. Chem..

[19] Carvalho S., Raposo A.C., Martins F.B., Grosso A.R., Sridhara S.C., Rino J., Carmo-Fonseca M., De Almeida S.F. (2013). Histone methyltransferase SETD2 coordinates FACT recruitment with nucleosome dynamics during transcription. Nucleic Acids Res..

[20] Kanu N., Grönroos E., Martinez P., Burrell R.A., Yi Goh X., Bartkova J., Maya-Mendoza A., Mistrík M., Rowan A.J., Patel H. (2015). SETD2 loss-of-function promotes renal cancer branched evolution through replication stress and impaired DNA repair. Oncogene.

[21] Cho Y.J., Liang P. (2008). Killin is a p53-regulated nuclear inhibitor of DNA synthesis. Proc. Natl. Acad. Sci. USA.

[22] Barrand S., Andersen I.S., Collas P. (2010). Promoter-exon relationship of H3 lysine 9, 27, 36 and 79 methylation on pluripotency-associated genes. Biochem. Biophys. Res. Commun..

[23] Dima M., Pecce V., Biffoni M., Di Gioia C.R.T., Tallini G., Biffoni M., Rosignolo F., Verrienti A., Sponziello M., Damante G. (2015). Molecular profiles of cancer stem-like cell populations in aggressive thyroid cancers. Endocrine.

[24] Khandrika L., Kim F.J., Campagna A., Koul S., Meacham R.B., Koul H.K. (2008). Primary Culture and Characterization of Human Renal Inner Medullary Collecting Duct Epithelial Cells. J. Urol..

[25] Pecce V., Sponziello M., Damante G., Rosignolo F., Durante C., Lamartina L., Grani G., Russo D., di Gioia C.R., Filetti S. (2018). A synonymous RET substitution enhances the oncogenic effect of an in-cis missense mutation by increasing constitutive splicing efficiency. PLoS Genet..

[26] Sponziello M., Silvestri G., Verrienti A., Perna A., Rosignolo F., Brunelli C., Pecce V., Rossi E.D.E.D., Lombardi C.P.C.P., Durante C. (2018). A novel nonsense EIF1AX mutation identified in a thyroid nodule histologically diagnosed as oncocytic carcinoma. Endocrine.

[27] Verrienti A., Tallini G., Colato C., Boichard A., Checquolo S., Pecce V., Sponziello M., Rosignolo F., De Biase D., Rhoden K. (2016). RET mutation and increased angiogenesis in medullary thyroid carcinomas. Endocr. Relat. Cancer.

[28] Sponziello M., Lavarone E., Pegolo E., Di Loreto C., Puppin C., Russo M.A., Bruno R., Filetti S., Durante C., Russo D. (2013). Molecular differences between human thyroid follicular adenoma and carcinoma revealed by analysis of a murine model of thyroid cancer. Endocrinology.

[29] Foster J.M., Oumie A., Togneri F.S., Vasques F.R., Hau D., Taylor M., Tinkler-Hundal E., Southward K., Medlow P., McGreeghan-Crosby K. (2015). Cross-laboratory validation of the OncoScan^®^ FFPE Assay, a multiplex tool for whole genome tumour profiling. BMC Med. Genom..

[30] Kruger N.J., Walker J.M. (2009). The Bradford Method For Protein Quantitation. The Protein Protocols Handbook.

[31] Mezi S., Chiappetta C., Carletti R., Nardini A., Cortesi E., Orsi E., Piesco G., Di Gioia C. (2017). Clinical significance of epithelial-to-mesenchymal transition in laryngeal carcinoma: Its role in the different subsites. Head Neck.

[32] Pfister S.X., Markkanen E., Jiang Y., Sarkar S., Woodcock M., Orlando G., Mavrommati I., Pai C.C., Zalmas L.P., Drobnitzky N. (2015). Inhibiting WEE1 Selectively Kills Histone H3K36me3-Deficient Cancers by dNTP Starvation. Cancer Cell.

