# Supplementary figures and images for: Loss of Function SETD2 Mutations in Poorly Differentiated Metastases from Two Hürthle Cell Carcinomas of the Thyroid
Source: Cancers (Basel). 2020 Jul 14;12(7):1892. doi: 10.3390/cancers12071892 (PMC7409075; doi:10.3390/cancers12071892)

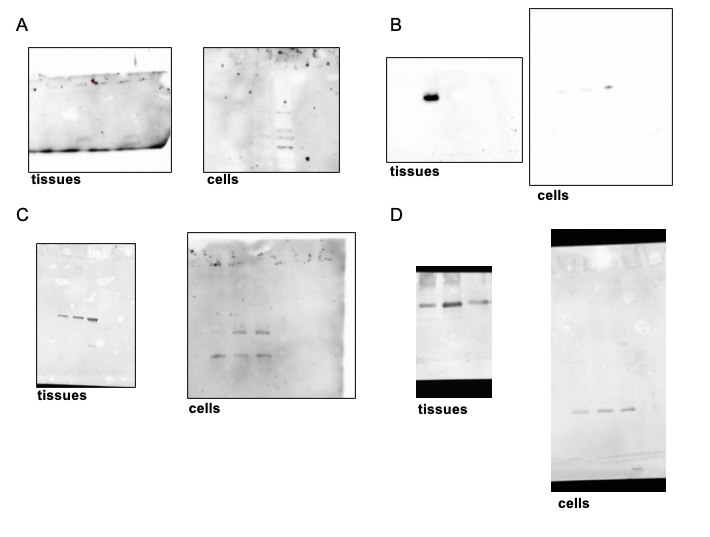

Supplement: Supplementary file 1 [file cancers-12-01892-s001.zip › Supplementary Files/SuppFig1.jpg]

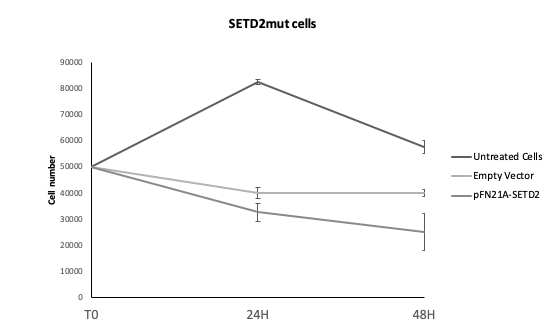

Supplement: Supplementary file 1 [file cancers-12-01892-s001.zip › Supplementary Files/SuppFig2.jpg]

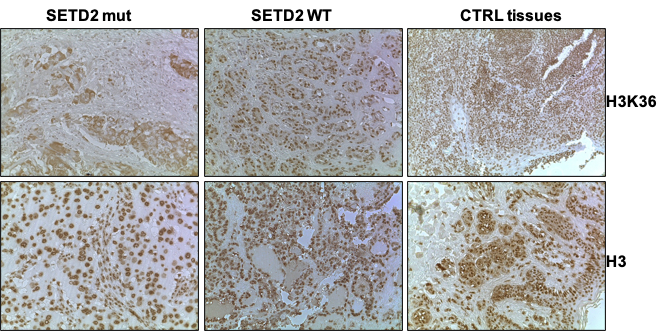

Supplement: Supplementary file 1 [file cancers-12-01892-s001.zip › Supplementary Files/SuppFig3.jpg]

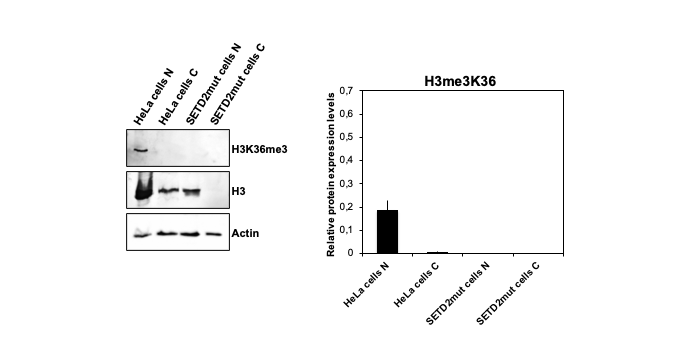

Supplement: Supplementary file 1 [file cancers-12-01892-s001.zip › Supplementary Files/SuppFig4.jpg]

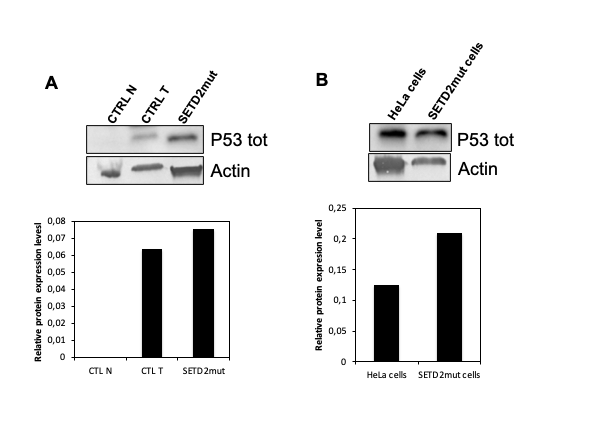

Supplement: Supplementary file 1 [file cancers-12-01892-s001.zip › Supplementary Files/SuppFig5.jpg]
